# From Stress to Neurodegeneration: A New Look at the Pathogenesis of Parkinson’s Disease

**DOI:** 10.3390/biomedicines14051130

**Published:** 2026-05-16

**Authors:** Rogneda B. Kazanskaya, Vassiliy Tsytsarev, Anna B. Volnova, Raul R. Gainetdinov, Alexander V. Lopachev

**Affiliations:** 1 Institute of Translational Biomedicine, St. Petersburg State University, Universitetskaya Nab. 7/9, 199034 St. Petersburg, Russia; a.volnova@spbu.ru (A.B.V.); r.gainetdinov@spbu.ru (R.R.G.); a.lopachev@spbu.ru (A.V.L.); 2 School of Engineering, Johns Hopkins University, Baltimore, MD 21218, USA; 3 Center for Transgenesis and Genome Editing, St. Petersburg State University, Universitetskaya Nab. 7/9, 199034 St. Petersburg, Russia

**Keywords:** Parkinson’s disease, stress, post-traumatic stress disorder, neuroinflammation, neurodegeneration

## Abstract

The relationship between stress and Parkinson’s disease is regarded as complex and multifaceted, although a direct causal link has not yet been conclusively proven. One prevailing hypothesis is based on the activation of the hypothalamic–pituitary–adrenal (HPA) axis and the consequent elevation of glucocorticoid levels. Prolonged exposure to these hormones may exacerbate oxidative stress, thereby rendering the dopaminergic neurons within the brain’s subcortical structures more susceptible to degeneration. Furthermore, stress may intensify neuroinflammation through the activation of microglia—a mechanism that could constitute a significant factor in the pathogenesis of Parkinson’s disease. Another important concept concerns the direct interaction of stressors with the dopaminergic system. Physiological and psychological stress can alter dopaminergic transmission by affecting both the synthesis and release of dopamine, as well as the sensitivity of dopamine receptors. Severe or chronic stress may contribute to the disruption of dopaminergic mechanisms and accelerate the onset of clinical symptoms in predisposed individuals. Furthermore, many researchers draw attention to the role of stress-induced aggregation of α-synuclein—a key protein implicated in the pathogenesis of Parkinson’s disease. Clinical data suggest a highly probable link between post-traumatic stress disorder and an increased risk of developing Parkinson’s disease, although these findings remain inconclusive. It is possible that stress acts not as a primary cause, but rather as a modifying factor that interacts with genetic predisposition, accelerating or triggering neurodegenerative processes. The aim of our narrative review was to examine these concepts and discuss possible directions for future research into the interaction between stress and Parkinson’s disease.

## 1. Introduction

The negative impact of psychological stress on physical and mental health has been understood ever since the 1980s [1,2]. The presence of chronic stress appears to significantly increase the risk of developing certain psychiatric illnesses, such as depression, bipolar disorder and post-traumatic stress disorder [3]. At the same time, advances in healthcare quality have greatly extended life expectancy in developed countries. In conjunction, these two factors contribute to the observed rise in the prevalence of neurodegenerative diseases. Parkinson’s disease, a debilitating neurodegenerative pathology characterized by dopaminergic neuron loss [4], is the second most frequent after Alzheimer’s disease. Only around 5–10% of all cases of Parkinson’s disease are genetic [4,5]—however, the mechanisms behind idiopathic Parkinson’s disease remain elusive. Parkinson’s disease is generally diagnosed only when around 30% of dopaminergic neurons in the substantia nigra have already been lost [6], and is characterized by an extensive prodromal stage that can last for decades [7]. There is no known cure for neurodegeneration, although recent studies in cell therapy show encouraging results [8]. The therapeutic approaches to treating Parkinson’s disease that do exist are aimed at increasing the patient’s quality of life, rather than addressing the core issue. It follows that targeting the prodromal phase of Parkinson’s disease is key to decreasing its prevalence. However, biomarkers that could facilitate early diagnosis are sparse and not highly specific [9]. This makes preventative treatment—and the development of such—extremely difficult.

To synthesize the available data on these issues, we conducted a literature search, primarily utilizing the PubMed database. We searched for peer-reviewed articles using combinations of relevant keywords, including Parkinson’s disease, stress, PTSD, neurodegeneration, loss of dopaminergic neurons, and other related terms as appropriate to identify publications addressing the role of stress in the development and progression of Parkinson’s disease. Additional references were identified through manual screening of cited literature in relevant articles.

Earlier review papers have attempted to associate chronic psychological stress with Parkinson’s disease [10]. Previously, Dallé et al. have suggested that there is a link between early-life psychological stress, depression, and Parkinson’s disease development [11]. We extend this association to chronic psychological stress across the lifespan. In this review, we argue that chronic psychological stress can be a facilitator, aggravator [12] and part of the premotor stage of Parkinson’s disease. It should also be noted that the prodromal stage of Parkinson’s disease is highly heterogeneous [13], possibly with distinct subtypes [14]. As such, we will discuss several psychological stress-triggered mechanisms that can lead to the development of some of these subtypes. Specifically, we focus on three interconnected biological mechanisms: (i) hypothalamic–pituitary–adrenal (HPA) axis dysregulation and glucocorticoid toxicity; (ii) neuroinflammation; and (iii) cellular stress responses, including oxidative stress and mitochondrial dysfunction. We also examine some of the under-represented molecular targets in prospective Parkinson’s disease treatment, including Na^+^,K^+^-ATPase and receptors associated with trace amines.

## 2. Psychological Stress and Parkinson’s Disease—What Is the Connection?

The incidence of Parkinson’s disease is relatively low, affecting 1% of the population above the age of 65 [15,16], and increasing to 4–10% at age 70 and older [17]. The number of people experiencing chronic stress, meanwhile, is much higher. This poses a valid question—how reasonable is it to suggest that chronic stress is one of the main players in the pathogenesis of idiopathic Parkinson’s disease? The epidemiology of Parkinson’s disease does not provide a definitive answer regarding the role of stress in the development of the disease. However, indirect evidence suggests that the incidence of PD is influenced by social activity, not solely by chemical or physical environmental exposures [18]. Because social activity modulates psychological stress, this finding is consistent with the hypothesis that stress-related mechanisms—in particular, dysregulation of the hypothalamic–pituitary–adrenal (HPA) axis—may play a role in PD pathogenesis, even if the absolute risk attributable to stress remains modest [19,20,21]. However, as will be discussed below, the direction of HPA axis change is not uniform: chronic stress can lead to either HPA hyperactivity or, paradoxically, HPA suppression depending on the nature of the stressor, individual vulnerability, and the presence of conditions such as post-traumatic stress disorder (PTSD) [22,23].

Reactions to stress vary considerably with sex, genetic background, and prior life experience [24]. Acute stress may have adaptive effects [25,26], whereas chronic stress is consistently implicated in the pathogenesis of psychiatric and neurodegenerative disorders [27,28]. As a whole, psychological stress has been shown to alter and impair cognitive function, as well as increase risk of mental health issues [29].

A century ago, it was noted that chronic stress and emotional trauma were often among the first hypothesized causes of Parkinson’s disease [30]. Modern studies also conclude that chronic stress may influence susceptibility to, or exacerbate the symptoms of, Parkinson’s disease through mechanisms involving neuroinflammation and dopamine depletion [10,31].

Therefore, stress was among the first proposed etiologies for PD, and a substantial body of correlative evidence now supports an association [10,32]. However, establishing causality requires an evaluation of specific interrelated pathways, which is the focus of this section.

Early-life psychological stress, which is associated with a number of mental illnesses [33], can cause decreased dopamine synthesis and dopamine depletion in the brain via HPA axis activation [32,34,35,36]. In animal models, chronic administration of corticosterone—the rodent equivalent of human cortisol—exacerbates 1-methyl-4-phenyl-1,2,3,6-tetrahydropyridine (MPTP)-induced dopaminergic cell death and increases susceptibility to α-synuclein pathology [37]. Human studies similarly show elevated cortisol in PD patients, which correlate with depressive symptoms and motor severity [38]. The impact of psychological stress on the progression of PD has been extensively reviewed by several research groups [39]. Taken together, these findings establish HPA axis hyperactivity as a plausible mechanism linking stress to PD, with a moderate level of evidence for association but still weak support for causality in human PD, as most data remain cross-sectional. Thus, HPA axis dysfunction is best understood as a likely disease-modifying or aggravating factor rather than a sole cause.

However, the relationship between the HPA axis and PD risk is not unidirectional. Post-traumatic stress disorder (PTSD), a condition defined by chronic psychological stress, is associated with a nearly two-fold increased risk of developing PD [40]. Paradoxically, PTSD is typically characterized by low basal cortisol levels and a blunted HPA axis response [41]. A leading mechanistic model resolves this paradox by proposing that enhanced glucocorticoid receptor sensitivity in PTSD induces a suppressive state, resulting in hypocortisolism despite ongoing psychological distress [41]. This pattern of HPA axis hypoactivity is also observed in certain atypical depression subtypes [42], which further demonstrates that chronic stress can produce divergent neuroendocrine profiles depending on individual genetic background, trauma history, and illness stage. Importantly, any sustained deviation in HPA axis activity from homeostatic set points—whether hyperactive or hypoactive—may disrupt central monoamine [32] and inflammatory signaling [43], thereby lowering the threshold for α-synuclein pathology [44] and accelerating disease progression.

One direct consequence of sustained HPA axis dysregulation, whether hyper- or hypoactive, is an increased vulnerability to major depressive disorder (MDD). Chronic psychological stress is a well-established risk factor for MDD [28,45], and MDD itself is both a prodromal symptom and a risk factor for PD [11,46]. In this way, depression can act as a mediator linking chronic stress to PD. Adverse childhood experiences, a form of early-life stress, reduce responsiveness to antidepressants and are associated with persistent monoaminergic alterations [47]. Animal models of chronic unpredictable mild stress (CUMS), which is considered to mimic socio-economic stress experienced by humans [48,49,50], induce depressive-like states and, when combined with low-dose neurotoxins, can unmask parkinsonian deficits [51,52]. The evidence for the stress–depression link is strong, while the depression–PD link is moderate, supported by meta-analyses [53] but with residual concerns about reverse causation (subclinical PD causing depression). Overall, depression likely lies on the causal pathway between chronic stress and PD, amplifying the neurobiological consequences of HPA dysregulation.

On the tissue level, chronic stress also promotes neuroinflammation and microglial priming, which constitute a third plausible mechanism linking stress to PD. CUMS and chronic restraint stress activate microglia and increase pro-inflammatory cytokines in animal models [51]. In MPTP and α-synuclein A53T mouse models, prior chronic stress worsens nigrostriatal degeneration and accelerates α-synuclein aggregation [52,54]. Studies have identified neuroinflammation and early neurodegeneration in subsets of patients with MDD [55,56], suggesting that stress-induced inflammatory processes may operate both independently and together with depressive pathology. Because neuroinflammation is a recognized PD risk factor [57], stress-induced microglial activation could lower the threshold for α-synuclein-triggered neurodegeneration. The experimental evidence from animal models is strong, but direct clinical evidence that stress-induced neuroinflammation precedes PD diagnosis remains weak to moderate. Consequently, this pathway is best understood as a permissive mechanism rather than an independent disease initiator.

A fourth pathway through which chronic stress may influence PD risk involves sleep disruption and consequent glymphatic dysfunction. Psychological stress frequently disrupts sleep architecture, and REM sleep behavior disorder (RBD) is a strong prodromal marker of PD [58]. Post-traumatic stress disorder (PTSD), which is characterized by stress-induced sleep fragmentation and hyperarousal, is associated with increased PD risk [59,60,61]. In transgenic PD mice, chronic sleep fragmentation accelerates α-synuclein accumulation and the emergence of prodromal symptoms, although it does not induce pathology in wild-type mice [62]. Impaired glymphatic clearance of misfolded proteins during disturbed sleep has been proposed as a mechanistic link [63]. The evidence for RBD as a prodromal marker of PD is strong, but the direct link from stress-induced sleep disruption to PD causation remains weak, as no human longitudinal study has examined whether stress-related sleep disturbances predict incident PD independently of RBD. Thus, similar to neuroinflammation, sleep disruption likely contributes to pathology development in individuals already vulnerable (e.g., those with incidental α-synuclein pathology) rather than acting as an independent trigger.

In sum, the available evidence does not support the claim that chronic stress alone causes PD in otherwise healthy individuals. Instead, chronic psychological stress likely acts as a risk amplifier in individuals with pre-existing vulnerabilities (e.g., genetic susceptibility, early-life stress, environmental toxin exposure). While not itself a causal pathway, chronic stress exacerbates motor symptoms in established PD patients, and stress-reduction practices are associated with better prognosis [64,65]. This observation provides indirect support for the hypothesis that stress may similarly influence prodromal stages. The pathways discussed above—HPA axis dysregulation, depression, neuroinflammation, and sleep disruption—are not mutually exclusive and likely converge on common cellular mechanisms such as oxidative stress, mitochondrial dysfunction, and impaired protein clearance. A summary of the mechanisms overviewed in this section is provided in Table 1.

## 3. The Hypothalamic–Pituitary–Adrenal Axis as a Link Between Psychological Stress and Physiological Response

### 3.1. HPA Axis Activation in Response to Chronic Psychological Stress and Despair-like States

There is evidence supporting a connection between chronic HPA axis hyperactivation, elevated cortisol levels, and neurodegeneration [66,67]. High plasma cortisol levels correlate with lower cerebral metabolism [68], which is associated with neurodegeneration [69]. In vitro, corticosterone administration causes dopaminergic neuron death, and exacerbates MPP^+^ toxicity [70]. In mice, increased corticosterone levels have been shown to increase susceptibility to neurodegeneration in the presence of pathological alpha-synuclein [37], implying that HPA axis hyperactivity may serve as a risk factor for PD.

There is an implicit assumption that the HPA axis is the primary physiological respondent to psychological stress. Evidence from clinical studies suggests that psychological stress can trigger changes in the HPA axis in patients with de novo Parkinson’s disease [71]. However, there is still some dispute on whether or not the HPA axis is activated in response to psychological (subjective) stress. Higher levels of subjective perceived stress do correlate with lower cognitive performance [72], but are not always reflected in cortisol-level changes [73,74]. Investigations of HPA axis activation in response to phobic fear exposure appear to indicate that the loss of control in a situation perceived as stressful leads to HPA axis activation [75], rather than the stressor itself. Loss of control has been conceptually linked to despair-like states in animal models [76], induced through prolonged exposure to situations of complete or partial loss of control, such as immobilization [77] and forced swimming [78]. Chronic unpredictable stress models [79] have been shown to cause persistent HPA axis hyperactivity [80], and direct corticosterone administration reproduces depressive behaviors in rodents [81]. This implies that despair-like states, rather than psychological stress on its own, may cause HPA axis activation.

However, our understanding of despair-like states and their evaluation in animal models remains incomplete [82]. Some studies report that chronic mild stress enhances cognitive function in rodents [83], and variability in stressor type, duration, and frequency across studies makes direct comparison difficult [82]. Moreover, learned helplessness—a core symptom of depression—can be mitigated by prior rescue experiences in animal models [84], suggesting that even within the same environment, outcomes depend on prior experiences. These observations underscore that individual differences in prior stress exposure, genetic background, and coping strategies moderate the relationship between stress and HPA alterations; not all individuals exposed to uncontrollable stress develop persistent dysregulation. At the same time, it implies that depression in conjunction with feelings of hopelessness and loss of control can form a positive feedback loop, decreasing the resistance to further emotional distress and increasing HPA axis activity.

These findings suggest that unpredictability and perceived loss of control—rather than stressor intensity alone—may be key determinants of HPA dysregulation. However, individual differences in coping, prior stress exposure, and genetic factors moderate this relationship, and not all individuals exposed to uncontrollable stress develop persistent HPA alterations.

### 3.2. The Effects of Prolonged HPA Axis Dysregulation in Conjunction with Chronic Stress on the Brain

It is widely known that chronic stress is a risk factor for MDD. Stress and depression often form a cycle, with stress triggering depression and vice versa. Traumatic events can be powerful triggers for depressive episodes. However, while stress is experienced by many, it more often leads to depression in people with pre-existing psychological vulnerabilities. It should also be mentioned that depression is a frequent comorbidity of PD [85], presumably due to elevated cortisol levels in PD patients [38].

It is important to acknowledge, however, that the relationship between depression and HPA axis activity is not uniform. Meta-analyses have consistently demonstrated that while many depressed patients exhibit HPA axis hyperactivity and elevated cortisol levels [86,87], this pattern is not universal. Blunted cortisol responses—particularly a flattened cortisol awakening response—are also observed, especially in milder or atypical depressive presentations, as well as in association with early-life trauma and chronic stress exposure [88]. This heterogeneity is partly accounted for by depression subtype: melancholic depression is more consistently linked to HPA axis hyperactivity, whereas atypical depression is more often characterized by normal or blunted cortisol output [42]. Thus, the direction and magnitude of HPA axis dysregulation in depression depend critically on clinical subtype, illness stage, and prior stress history. In Parkinson’s disease, available evidence predominantly points toward HPA axis hyperactivity, with elevated cortisol levels reported in PD cohorts and associated with greater motor impairment and depressive symptoms [20,38]. However, longitudinal studies examining whether blunted cortisol responses occur in specific subgroups of PD (e.g., early-stage disease, atypical presentations) remain lacking, representing an important gap for future research.

With this heterogeneity in mind, how does HPA axis dysfunction in Parkinson’s disease compare to that observed in depression? As mentioned above, adverse childhood experiences are associated with HPA axis function alterations [47]. Stress can induce increases in cortisol levels that are associated with higher vulnerability to depression in animal models [89,90], and HPA axis hyperactivity is observed in most depressed patients [86]. At the same time, studies have shown that in depression, more corticotropin-releasing hormone is released from the hypothalamus [91], leading to chronic hyperactivation of the HPA axis [92,93]. Reducing HPA axis activity using retinoic acid receptor alpha agonists in models of stress-induced depression ameliorates the symptoms [94].

In the brain, one of the regions most densely populated with corticosteroid receptors is the hippocampus [95]. It follows that the hippocampus is heavily affected by HPA axis hyperactivity. In patients with depression [96] and in chronic stress-induced depression models [97], higher levels of cortisol (exogenous or endogenous) correlate with better recall and memory formation, as well as altered hippocampus signaling in females. This pattern is not characteristic of healthy individuals.

At the same time, high levels of cortisol appear to be toxic to hippocampus neurons [98,99]. It is also known that glucocorticoids can cause cellular oxidative stress via glucocorticoid-responsive elements and increased cellular metabolism, leading to higher reactive oxygen species generation [100]. However, in rats, chronic injection of corticosterone over the course of 60 days resulted in decreased synapse number in the hippocampus [101], but did not cause neuron loss.

There are studies indicating elevated cortisol levels correlate with a decrease in hippocampal volume in depressed patients [102,103]. PTSD patients also display lower hippocampal volume in comparison to individuals who have not experienced trauma followed by chronic stress [104]. At the same time, in rodent models, CUMS-induced depression is accompanied by a decrease in hippocampal volume [105,106,107] and higher corticosterone levels [108]. However, it should be mentioned that this correlation should be treated with caution, despite support from animal models, as smaller hippocampal volume may precede and increase vulnerability to stress and trauma [109], rather than being solely a consequence of cortisol excess.

These findings suggest a bidirectional relationship: stress and HPA axis activation can reduce hippocampal volume, and reduced hippocampal volume impairs negative feedback to the HPA axis, perpetuating further activation [110]. This feedback loop may have a ceiling effect, because severe stress-induced hippocampal damage can also downregulate glucocorticoid receptor expression, leading to a blunted stress response [111].

How do these hippocampal changes relate to PD? Some authors suggest that decreased hippocampal volume may be a precursor of amyloidosis [66], while others indicate that while hippocampal neurons are not as affected by neurodegeneration in PD, changes at the synaptic level and in response to alpha-synuclein are observed in them [112]. Beyond the hippocampus, PD involves stress-sensitive circuits, including the amygdala, prefrontal cortex, and striatum—regions where glucocorticoid receptor expression and HPA coupling remain underexplored.

For further information on the role of the HPA axis in depression outside of the context of PD, we refer the reader to a review by Bao and Swaab [113].

### 3.3. Dysregulated Norepinephrine Signaling in Response to HPA Axis Hyperactivity

Unlike the slower HPA axis, the sympatho–adrenal–medullary (SAM) axis provides an immediate, neuron-mediated response to stress [114]. The brain sends signals via the sympathetic nervous system to preganglionic nerve fibers, which release acetylcholine and stimulate the adrenal medulla, leading to the release of the catecholamines adrenaline and norepinephrine into the bloodstream. These hormones trigger increased heart rate, elevated blood pressure, and a rise in blood glucose levels. In PD, the SAM axis is affected by neurodegeneration, resulting in decreased catecholamine production in the adrenal medulla. Clinically, this dysfunction typically manifests as autonomic failure, including hypotension and a blunted stress response. In PD, Lewy bodies (aggregates of α-synuclein) are found in the sympathetic ganglia and adrenal medulla. The SAM axis plays a key role in stress, but it remains unclear whether components of this axis are affected in PD independently of stress, or whether they themselves participate in the development of PD [77,115].

The norepinephrine system—central to both SAM axis output and central stress regulation—plays an important role in PD (reviewed in [116,117]), with degeneration of noradrenergic neurons in the locus coeruleus [118] exceeding and preceding the loss of dopaminergic neurons [119,120,121]. Degeneration of the locus coeruleus, accompanied by reduced norepinephrine levels, contributes to cognitive and motor deficits in Parkinson’s disease, but is not addressed in pharmacological treatments [122]. This is supported by the fact that dopamine β-hydroxylase knockout mice lacking norepinephrine exhibit pronounced deficits in motor and coordination abilities [123].

Norepinephrine (NE) is a key neuromodulator within the monoaminergic system, playing a pivotal role in the regulation of attention, arousal, and cognitive processes. By interacting with the serotonergic and dopaminergic systems, it optimizes cortical function and synaptic plasticity. Abnormal NE transmission is associated with various pathologies, including neurodegenerative diseases. Norepinephrinergic neurons in the locus coeruleus innervate dopaminergic neurons in the ventral tegmental area, which, in turn, innervate dopaminergic neurons in the nucleus accumbens [124,125]. Electrical stimulation of the locus coeruleus induces release of norepinephrine in the VTA and dopamine in the nucleus accumbens [126]. These effects are partly mediated through adrenergic receptors, including α2C-AR [127], which regulate not only norepinephrine transmission but also dopamine, serotonin, and GABA signaling [128,129] in a number of brain regions affected in Parkinson’s disease, including the locus coeruleus [126], ventral tegmental area [130], nucleus accumbens [131,132], and substantia nigra [127].

In addition to its direct role in modulating dopaminergic and serotoninergic transmission, norepinephrine shows neuroprotective properties in physiological concentrations, reducing both excitotoxic and inflammatory responses, as well as oxidative stress damage. Several studies demonstrate the neuroprotective properties of norepinephrine in MPTP-induced parkinsonism models [133,134]. These effects of norepinephrine presumably involve induction of TrkB:BDNF signaling [135]. BDNF is a neuroprotective transcription factor that has been shown to alleviate depressive symptoms in chronically stressed mice [136]. For an in-depth look at the neuroprotective properties of norepinephrine, we suggest a review by Feinstain et al. [137].

The role of the locus coeruleus in stress response has been extensively reviewed by Ross et al. [138]. It also plays an important role in the response to CUMS and modulates the HPA axis [80]. CUMS reduces norepinephrine release from the locus coeruleus [139], antidepressants targeting norepinephrinergic transmission have been shown to improve monoaminergic tone in CUMS-affected rats [140], and chronic social stress has been shown to upregulate norepinephrine transporter expression in rats [141]. These findings are supported by clinical investigations. Dysregulation of norepinephrine signaling is linked to the development of stress-induced MDD [142], and reduced levels of norepinephrine transporters were observed in the locus coeruleus of such patients [143].

Importantly, in the context of PD, we cannot determine whether the LC plays a role in triggering the mechanism that leads to the development of Parkinson’s disease. However, under conditions of stress, a compromised noradrenergic system is unable to adequately regulate arousal, leading to a disproportionate worsening of both the neurological and psychiatric symptoms of Parkinson’s disease [144].

Although it remains unclear whether the locus coeruleus directly initiates PD pathology, it is consistently affected early in the disease [145], resulting in norepinephrine deficiency. Under stress conditions, this impairment may lower the threshold for symptom manifestation. Clinical observations describe cases where severe stress triggered the onset of PD symptoms [146], while animal studies show that stress can induce parkinsonian-like motor deficits in dopamine-depleted states [147].

As a whole, it can be concluded that chronic stress causes persistent dysregulation of HPA axis signaling, which induces changes in norepinephrine transmission. Due to norepinephrine’s key role in modulating monoaminergic transmission, its dysregulation can lead to various mood disorders, and is involved in PD.

## 4. Stress-Mediated Molecular and Cellular Mechanisms of Parkinsonism Development as Possible Therapeutic Targets

Parkinson’s disease is a multifactorial disorder. It is difficult to identify a single specific mechanism or factor that triggers or mediates the development of pathogenesis, unless mutations in specific genes occur in a small percentage of cases. It is also important to keep in mind that the initiating factors and mechanisms of PD pathogenesis, such as oxidative stress, protein aggregation, mitochondrial dysfunction, and disruptions in proteins key to neuronal function (such as Na^+^,K^+^-ATPase (NKA)), closely overlap with those of other neurodegenerative diseases. It is possible that chronic and physiological stress may act synergistically on several of these mechanisms.

### 4.1. Oxidative Stress

Oxidative stress is a well-established contributor to neurodegeneration, and neurons—particularly dopaminergic neurons of the substantia nigra—are exceptionally vulnerable due to their high metabolic rate, calcium oscillations, and reliance on reactive oxygen species (ROS) for signaling [148,149,150,151,152]. Chronic psychological stress has been shown to increase oxidative stress in brain regions implicated in both depression and PD [153]. In animal models, variable or unpredictable chronic stress activates oxidative stress response genes [154], and the underlying mechanisms are thought to involve HPA axis activation and stress-related hormones [100,155]. Although the precise molecular link between psychological stress and ROS generation remains incompletely understood, the evidence for an association is consistent across multiple rodent models [156], and it is possible that they are mediated by activation of the hypothalamic–pituitary–adrenal axis [100] and stress-related hormones [155], as discussed above.

Oxidative stress has been shown to be present in the pathogenesis of MDD, with some authors even suggesting the use of antioxidants in treating it (reviewed in [157]). Depression is also implied to be a neurodegenerative disorder, with the oxidative and nitrosative pathways being more active while the overall antioxidant status is decreased in patients and animal models [158]. Elevated oxidative stress levels have been observed in oligodendrocytes of depressed patients [159].

It is known that myelin-producing oligodendrocytes maintain the highest iron concentrations in the brain [160,161], suggesting that they are the most sensitive to disruptions in iron homeostasis. Gene expression and brain iron are reviewed in [162]. For more information on the interplay between iron and protein misfolding in PD, please refer to [163]. The authors hypothesize that a vicious cycle involving oxidative stress, protein misfolding and the interaction between beta-amyloid and iron plays a key role in the pathogenesis of Parkinson’s disease.

The brain is extremely sensitive to oxidative stress, due to both low antioxidant levels and high reactive oxygen species production by microglia [164] and neurons [165]. The presence of significant amounts of iron exacerbates oxidation and can lead to ferroptosis [166]. Both the locus coeruleus and substantia nigra accumulate neuromelanin, a compound that serves a dual purpose—storing excess dopamine as a stable compound, and chelating iron. Iron is present in the brain in high concentrations due to its involvement in ATP generation [167,168], catecholamine synthesis [169], neurotransmitter metabolism [170], and myelination.

To a certain point, this is an effective way of protecting catecholamine neurons from ferroptosis and oxidative stress. However, recent studies show that neuromelanin may contribute to neurodegeneration via microglia activation [171,172,173].

Prolonged severe stress (2 h immobilization for 3 days) in mice caused increased dopamine and tetrahydrobiopterin (a dopamine synthesis cofactor that can undergo auto-oxidation to produce hydrogen peroxide and superoxide radical [174] synthesis, leading to neurodegeneration in the nigrostriatal system [175]). Since neuromelanin synthesis is increased in response to excess catecholamines in the cytoplasm [176], dysregulation of dopamine metabolism can lead to an increase in neuromelanin synthesis, increasing the risk of oxidative stress down the line.

A recent study has shown that more neuromelanin is present in neurons with less vesicular monoamine transporter 2 (VMAT-2) [177], further confirming the correlation between dopamine concentrations and neuromelanin. The role of neuromelanin in neurodegenerative processes has been reviewed extensively by Zucca et al. [178]. A recent proteomic study indicates that neuromelanin granules are associated with stress granules, and may be derived from them [179].

Neuromelanin is one of the mediators in translating oxidative stress into neuroinflammation via its excitatory effects on microglia, both protecting neurons and contributing to a vicious cycle of neurodegeneration and inflammation [180]. A recent study suggests that targeting iron deposition in nigrosomes could be an effective treatment for stalling PD progression [181].

The efficacy of various antioxidant molecules, including vitamins, polyphenols, flavonoids, and others, has been demonstrated in various models of PD [182,183]. However, the therapeutic potential of such agents is very limited [184], and interventions targeting other pathogenesis mechanisms are required.

### 4.2. Glia Involvement in the Development of Neurodegeneration

Over the past several years, data supporting the neuromodulatory role of astrocytes in pathological conditions has accumulated [185]. A study has shown that grafting healthy astrocytes alleviated nigrostriatal toxicity in MPTP-induced parkinsonism in mice via a Nrf2-driven Wnt/β-catenin prosurvival axis [186]. At the same time, inhibiting the angiotensin II type 1 receptor in astrocytes improved dopamine turnover and locomotion in an animal model of Parkinson’s disease [187], while also increasing BDNF and GDNF expression [188,189]. Nrf2 is a transcription factor involved in antioxidant defenses, and is implicated in suppressing ferroptosis and mitochondrial dysfunction [190]. The role of Nrf2 in neurodegenerative disorders is reviewed extensively in [191].

It should be noted that synaptic impairments precede neuronal degeneration in Parkinson’s disease, with one of the mechanisms implicated in synaptic loss being impaired astrocyte function [192]. The molecular mechanisms behind synaptic impairments observed in the pathogenesis of Parkinson’s disease have recently been reviewed by Gcwensa et al. [193]. Dopamine metabolites can cause dysregulation of mitochondrial function in astrocytes [194]. The role of astrocytes in neurodegenerative pathologies is further explored in a recent review by Bouvier et al. [195]. The role of glia as a whole in neuromodulation during neurodegenerative processes was reviewed extensively by Stevenson et al. [196].

### 4.3. Possible Role of Neuroinflammation

Stress experienced in childhood has been linked to chronic neuroinflammation and a higher risk of and poorer prognosis for PD [11,197]. Chronic stress is associated with neuroinflammatory processes, which are in turn associated with PD progression. More specifically, glucocorticoids play a dual role in inflammation—historically, they have been associated with anti-inflammatory hormones, but in certain conditions may promote neuroinflammation and microglia activation, which is implicated in PD [198]. Patients with PD have been shown to have elevated glucocorticoid levels [199]. Chronic stress was shown to exacerbate glucocorticoid-dependent inflammatory response and neurodegeneration in rats [200]. The role of microglia activation and neuroinflammation in Parkinson’s disease has been reviewed by Herrera et al. [198].

An inflammation and neurodegeneration hypothesis of depression has been proposed [158,201], suggesting that neuroinflammation is a key player in depression pathogenesis. A number of antidepressants have been shown to possess anti-inflammatory activity in the treatment of MDD [202]. It is plausible that the neurodegenerative processes that are a hallmark of PD could be a continuation of the ones observed in depression.

### 4.4. Na^+^, K^+^-ATPase as an Element in the Pathogenesis of PD and Possible Therapeutic Target

Oxidative stress or other mentioned pathogenic mechanisms do not act in isolation; they impair specific proteins that maintain neuronal integrity. One such critical target is the α3 subunit of Na^+^, K^+^-ATPase (NKA), which is expressed exclusively in neurons and is essential for rapidly restoring ionic gradients after action potentials [181]. In the central nervous system (CNS), NKA is represented by three isoforms: α1 is present in all cells and is responsible for maintaining the Na^+^ and K^+^ gradient, α2 is present in glial cells, and α3 is present exclusively in neurons [203]. Moreover, different neurons have different ratios of α1 and α3. Since the α3 isoform is necessary for rapidly restoring the Na^+^ and K^+^ gradient following an action potential, α3 is more abundant in neurons that generate action potentials frequently, such as fast-spiking interneurons. Disruptions in α3 function are known to be associated with the development of neurological and neuropsychiatric pathologies, such as rapid-onset dystonia, parkinsonism (RDP), alternate hemiplegia of childhood (AHC), bipolar disorder, and depression [204]. Partial dysfunction of the α3 isoform of NKA in the presence of mutations, as in the case of RDP, does not lead to the development of dopaminergic neuron degeneration [204]. However, this does not mean that NKA dysfunction is not involved in the pathogenesis of classical PD. Many experimental studies on laboratory animals have shown that chronic stress causes a decrease in NKA activity [205,206]. Pathogenic factors arising from chronic stress, such as oxidative stress, aggregates of misfolded proteins (α-syn, β-am, SOD), and neuroinflammation, also cause a decline in α3 NKA activity. How oxidative stress and aggregates of misfolded proteins cause a decrease in NKA activity has been described in detail in experimental articles and reviews [207,208,209,210]. In turn, α3 NKA dysfunction can increase the sensitivity of animals to the effects of OS. Chronic stress has been shown to induce depression to a greater extent in animals with a heterozygous α3 NKA knockout than in wild-type animals [211].

In terms of the effect of cortisol/corticosterone on NKA activity, there are a number of studies that describe that corticosterone can cause an increase in NKA activity, including in synaptosomes and brain homogenates [212], as well as an increase in the expression of mRNA isoforms of NKA in the heart under the action of glucocorticoids [213]. At the same time, in the model on erythrocytes, as well as the choroid plexus, a decrease in NKA activity in response to cortisol was shown [212,214].

Thus, disruption of NKA function, particularly the α3 isoform in the brain, can be considered an element of the pathogenic cascades that develop as a result of chronic stress and lead to pathological conditions in the central nervous system, including neurodegeneration. Restoring normal NKA function in the central nervous system through pharmacological interventions may be one option for reversing the negative consequences of chronic stress.

Cardiotonic steroids (CTSs) can be used to target NKA. It has been shown that low (nanomolar and below) concentrations of CTS, NKA ligands, can have anti-inflammatory properties, reduce the aggregation of misfolded proteins, and exert a neuroprotective effect in ischemic models [215,216,217,218]. A serious limiting factor in the potential use of CTS for the treatment of neurodegenerative diseases is their systemic effects on the cardiovascular system and other body systems [219]. A solution could be targeted delivery of CTS to the brain or the development of molecules that specifically bind only to the α3 isoform of NKA.

### 4.5. Trace Amine-Associated Receptors as an Emerging Therapeutic Target

One pharmacological class targeting Parkinson’s disease is catechol-O-methyltransferase (COMT) inhibitors, which are recommended as a first-line adjunct to L-DOPA therapy. Tolcapone, for instance, is a well-established COMT inhibitor that prevents peripheral L-DOPA metabolism, thereby increasing L-DOPA availability in the brain [220]. However, L-DOPA administration not only elevates striatal dopamine levels but also raises 3-methoxytyramine (3-MT) concentrations; tolcapone, conversely, reduces 3-MT levels [221]. How this central effect of COMT inhibitors contributes to their overall pharmacological profile remains to be elucidated [220].

Trace amine-associated receptors (TAARs) are G protein-coupled receptors. In humans, six functional TAAR subtypes have been identified [222]. Accumulating evidence indicates that TAARs significantly modulate the brain’s monoamine system and are involved in stress responses and emotional behaviors [223]. Recent studies have linked TAAR1 to neuroinflammation and TAAR5 to neurogenesis [224]. The best-characterized subtype, TAAR1, can be activated by 3-methoxytyramine (3-MT), a COMT-mediated extracellular dopamine metabolite, and this activation has been implicated in the generation of abnormal involuntary movements in mice [225].

Ulotaront, the first TAAR1 agonist to enter clinical testing, shows promise for treating schizophrenia. Importantly, clinical trials are currently evaluating its potential efficacy in Parkinson’s disease psychosis, generalized anxiety disorder, and MDD [226]. More broadly, TAAR1 agonists may represent a novel class of drugs for modulating anxiety [227], post-traumatic stress disorder manifestations [228], and mood disorders [229,230]. These stress-coping properties of TAAR1 agonists could meaningfully influence Parkinson’s disease pathogenesis. Supporting this possibility, recent studies have demonstrated that TAAR1 agonists protect neurons from oxidative stress [231] and show efficacy in models of parkinsonism and neuroinflammation [232,233].

## 5. Alternative Hypothesis: The Connection Between Parkinson’s Disease and PTSD

Individuals with PTSD have an increased risk of developing PD, but the increased risk is not very large—two times or less [16,40]. Even with comparable trauma exposure, only a subset of individuals develop PTSD, and among those who do, only a fraction later develop PD [234]. This suggests that there must be an intermediate mechanism between stress-related reactions and the neurodegenerative process that leads to PD.

PTSD occurs only in some people after similar traumas. It is believed to be influenced by genetic differences (likely in the dopaminergic and serotonergic systems and the HPA axis), differences in stress hormone regulation, and differences in the neuroinflammatory response [235]. In other words, PTSD can be viewed not simply as a consequence of stress, but also as an indicator of a specific type of neurobiological response to stress.

Several lines of evidence point to existing vulnerability as the critical factor that determines who develops PTSD after trauma and who, among those, goes on to develop PD. Genome-wide association studies have begun to map genetic risk, revealing striking overlaps with PD-related pathways. Notably, PARK2, a gene critical for dopamine regulation and a well-established cause of early-onset PD, has been robustly associated with PTSD [236]. Furthermore, a large-scale cross-trait genetic analysis reported considerable polygenic overlap between PTSD and neurodegenerative diseases, identifying 15 distinct shared genomic loci [236]. These findings support a model where shared genetic risk, particularly affecting the dopaminergic system, contributes to susceptibility for both PTSD and PD.

There are several biological pathways through which certain stress responses can accelerate neurodegeneration [67]. The first is the HPA axis and cortisol release. Some studies also show that chronic stress can increase the aggregation of alpha-synuclein, which is directly related to the pathogenesis of PD. Chronic hyperactivation of the stress system can lead to elevated cortisol levels and increased oxidative stress [20]. This may make dopamine neurons in the substantia nigra more vulnerable [20,237].

Second, PTSD increases cytokine release, which increases neuroinflammation and chronic microglial activation [238,239]. Neuroinflammation is known to be one of the key mechanisms in the progression of PD [240]. Some studies also show that chronic stress can increase alpha-synuclein aggregation, which is directly linked to the pathogenesis of PD [240,241].

It is also possible that PTSD and PD are partly based on the same individual characteristic of the dopamine system. Thus, PTSD could be an early marker of predisposition to Parkinson’s [40]. This hypothesis reverses the direction of causality. One line of research focuses on genetic predisposition to the development of PTSD following exposure to stress. Evidence suggests that PTSD is associated with alterations in systems involved in threat appraisal and the consolidation of traumatic memories [242,243].

Dopaminergic transmission plays an important role in reward processing, motivation, and reinforcement-based learning, including safety learning. For this reason, it may influence how strongly the brain “tags” a traumatic event as significant and how effectively this memory is retained [243,244]. Certain genetic variants related to dopaminergic function have been associated with increased stress reactivity, differences in emotion regulation, and enhanced fear learning [242].

This suggests that genetic factors do not directly cause PTSD, but rather increase sensitivity to stressful experiences. The same genetic variant may increase risk in adverse conditions, while having little or no negative effect in a supportive environment [243].

At the same time, PTSD is a multisystem condition. In addition to dopamine, other systems play key roles, including norepinephrine (involved in hyperarousal), serotonin (emotion regulation), and the hypothalamic–pituitary–adrenal (HPA) axis, which regulates the hormonal stress response The multicomponent nature of this system implies broad genetic diversity.

The usual assumption is stress → PTSD → neurodegeneration → Parkinson’s disease. However, an alternative pathway is possible: innate vulnerability of the dopamine system → (increased risk of developing PTSD and/or PD). That is, PTSD may be an early marker of the same vulnerability of the dopamine system that can later lead to the development of PD [40]. This hypothesis explains why not all individuals develop PTSD after trauma, why PTSD is associated with an increased risk of PD, and why stress can accelerate the onset of PD but is not the primary cause [61]. In other words, PTSD may not be a risk factor, but rather an early phenotypic indicator of dopamine system vulnerability.

## 6. Conclusions

The evidence reviewed here indicates that chronic psychological stress—particularly when characterized by perceived loss of control and helplessness—may contribute to Parkinson’s disease pathogenesis, but primarily as a disease modifier or risk amplifier rather than as a sole cause. The strongest support exists for pathways involving HPA axis dysregulation, depression as a mediator, neuroinflammation, sleep disruption, and exacerbation of motor symptoms. However, the available human evidence remains largely cross-sectional, and causal inference is limited by a lack of prospective studies linking premorbid stress-related biomarkers to incident PD. Therefore, while chronic stress may create pathogenetic prerequisites for PD in individuals with pre-existing vulnerabilities (e.g., genetic predisposition, incidental α-synuclein pathology, or early-life programming), it is neither necessary nor sufficient for disease development. Future longitudinal studies with repeated measures of stress, both psychological and physiological via biomarkers, and well-phenotyped PD outcomes are required to determine whether stress reduction strategies can modify long-term PD risk.

## Figures and Tables

**Table 1:** Summary of mechanistic pathways linking chronic psychological stress to Parkinson’s disease pathogenesis.

Proposed Pathway	Evidence Type	Direct vs. Indirect Mechanism	Summary of Key Evidence (from Section 2)	Strength of Evidence for Association	Support for Causality in Human PD
HPA Axis Dysregulation	Human & Preclinical	Direct (neuroendocrine)	Human: Elevated cortisol in PD correlates with depressive symptoms/motor severity; PTSD (blunted HPA) linked to 2x increased PD risk. Preclinical: Chronic corticosterone exacerbates MPTP-induced dopaminergic cell death & α-syn pathology.	Moderate (cross-sectional human data; consistent preclinical)	Weak (best understood as a disease-modifying/aggravating factor, not a sole cause. Direction of effect is inconsistent: hyperactivity vs. hypoactivity).
Depression (as a mediator between stress and PD)	Human & Preclinical	Indirect (psychiatric comorbidity)	Human: MDD is a prodromal symptom & risk factor for PD (meta-analyses support link). Chronic stress is a well-established risk factor for MDD. Preclinical: CUMS induces depressive-like states & exacerbates parkinsonian deficits when combined with neurotoxins.	Strong (stress leading to depression); moderate (depression leading to PD, with residual concern for reverse causation).	Moderate (likely lies on the causal pathway, amplifying HPA dysregulation, but subclinical PD causing depression is a competing explanation).
Neuroinflammation & Microglial Priming	Direct (cellular/immune)	Direct (cellular/immune)	Human: Neuroinflammation seen in subsets of MDD patients; recognized PD risk factor. Direct evidence linking stress-induced neuroinflammation to pre-diagnosis PD is lacking. Preclinical: CUMS/restraint stress activates microglia & increases cytokines; worsens nigrostriatal degeneration & accelerates α-syn aggregation in MPTP/A53T models.	Strong (preclinical); weak–moderate (direct clinical evidence)	Weak (best understood as a permissive mechanism that lowers the threshold for α-synuclein-triggered neurodegeneration, not an independent disease initiator).
Sleep Disruption & Glymphatic Dysfunction	Human & Preclinical	Indirect (physiological)	Human: RBD is a strong prodromal PD marker; PTSD (stress-induced sleep fragmentation) increases PD risk. No longitudinal study directly linking stress-related sleep disturbance to incident PD (independent of RBD). Preclinical: Chronic sleep fragmentation accelerates α-syn accumulation & prodromal symptoms in transgenic PD mice (but not in wild-type).	Strong (RBD leading to PD); weak (stress leading to sleep disturbances and directly to PD).	Weak (contributes to pathology in already vulnerable individuals; acts as a risk amplifier rather than an independent trigger).

## Data Availability

No new data were created or analyzed in this study. Data sharing is not applicable to this article.

## Abbreviations

The following abbreviations are used in this manuscript:

CUMS	Chronic unpredictable mild stress
PVN	Paraventricular nucleus of the hypothalamus
CRH	Corticotropin-releasing hormone
AVP	Arginine vasopressin
ACTH	Adrenocorticotropic hormone
HPA	Hypothalamic–pituitary–adrenal axis
SAM	Sympatho–adrenal–medullary axis
PD	Parkinson’s disease
MDD	Major depressive disorder
PTSD	Post-traumatic stress disorder
MPTP	1-methyl-4-phenyl-1,2,3,6-tetrahydropyridine
NKA	Na^+^,K^+^-ATPase
TAARs	Trace amine-associated receptors
3-MT	3-methoxytyramine
COMT	Catechol-O-methyltransferases

## References

[1] Gore J.C., Li M., Gao Y., Wu T.-L., Schilling K.G., Huang Y., Mishra A., Newton A.T., Rogers B.P., Chen L.M. (2019). Functional MRI and resting state connectivity in white matter—A mini-review. Magn. Reson. Imaging.

[2] DeLongis A., Folkman S., Lazarus R.S. (1988). The impact of daily stress on health and mood: Psychological and social resources as mediators. J. Personal. Soc. Psychol..

[3] Davis M.T., Holmes S.E., Pietrzak R.H., Esterlis I. (2017). Neurobiology of Chronic Stress-Related Psychiatric Disorders: Evidence from Molecular Imaging Studies. Chronic Stress.

[4] Klein C., Westenberger A. (2012). Genetics of Parkinson’s disease. Cold Spring Harb. Perspect. Med..

[5] Simon D.K., Tanner C.M., Brundin P. (2020). Parkinson Disease Epidemiology, Pathology, Genetics, and Pathophysiology. Clin. Geriatr. Med..

[6] Cheng H.C., Ulane C.M., Burke R.E. (2010). Clinical progression in Parkinson disease and the neurobiology of axons. Ann. Neurol..

[7] Mahlknecht P., Seppi K., Poewe W. (2015). The Concept of Prodromal Parkinson’s Disease. J. Park. Dis..

[8] Altarche-Xifro W., di Vicino U., Muñoz-Martin M.I., Bortolozzi A., Bové J., Vila M., Cosma M.P. (2016). Functional Rescue of Dopaminergic Neuron Loss in Parkinson’s Disease Mice After Transplantation of Hematopoietic Stem and Progenitor Cells. eBioMedicine.

[9] Li T., Le W. (2020). Biomarkers for Parkinson’s Disease: How Good Are They?. Neurosci. Bull..

[10] Djamshidian A., Lees A.J. (2014). Can stress trigger Parkinson’s disease?. J. Neurol. Neurosurg. Psychiatry.

[11] Dallé E., Mabandla M.V. (2018). Early Life Stress, Depression And Parkinson’s Disease: A New Approach. Mol. Brain.

[12] Johnson M.E., Stecher B., Labrie V., Brundin L., Brundin P. (2019). Triggers, Facilitators, and Aggravators: Redefining Parkinson’s Disease Pathogenesis. Trends Neurosci..

[13] Jankovic J., Tan E.K. (2020). Parkinson’s disease: Etiopathogenesis and treatment. J. Neurol. Neurosurg. Psychiatry.

[14] Berg D., Borghammer P., Fereshtehnejad S.M., Heinzel S., Horsager J., Schaeffer E., Postuma R.B. (2021). Prodromal Parkinson disease subtypes—Key to understanding heterogeneity. Nat. Rev. Neurol..

[15] de Lau L.M., Breteler M.M. (2006). Epidemiology of Parkinson’s disease. Lancet Neurol..

[16] Weaver F.M., Cao L., Stroupe K.T., Gonzalez B., Brown E., Colletta K., Tanner C.M., Goldman S.M. (2024). Post-Traumatic Stress Disorder and Risk of Parkinson’s Disease in a Veteran Cohort. J. Park. Dis..

[17] Hirsch L., Jette N., Frolkis A., Steeves T., Pringsheim T. (2016). The Incidence of Parkinson’s Disease: A Systematic Review and Meta-Analysis. Neuroepidemiology.

[18] Darweesh S.K.L., Ikram M.K., Faber M.J., de Vries N.M., Haaxma C.A., Hofman A., Koudstaal P.J., Bloem B.R., Ikram M.A. (2018). Professional occupation and the risk of Parkinson’s disease. Eur. J. Neurol..

[19] Polenick C.A., Birditt K.S., Turkelson A., Perbix E.A., Salwi S.M., Zarit S.H. (2020). Daily Social Interactions and HPA Axis Activity Among Midlife and Older Adults. Gerontologist.

[20] Knezevic E., Nenic K., Milanovic V., Knezevic N.N. (2023). The Role of Cortisol in Chronic Stress, Neurodegenerative Diseases, and Psychological Disorders. Cells.

[21] Goltz F., Van Der Heide A., Helmich R.C. (2024). Alleviating Stress in Parkinson’s Disease: Symptomatic Treatment, Disease Modification, or Both?. J. Park. Dis..

[22] Ring M. (2025). An Integrative Approach to HPA Axis Dysfunction: From Recognition to Recovery. Am. J. Med..

[23] Hou G., Tian R., Li J., Yuan T.F. (2013). Chronic Stress and Parkinson’s Disease. CNS Neurosci. Ther..

[24] Handa R.J., Sheng J.A., Castellanos E.A., Templeton H.N., McGivern R.F. (2022). Sex Differences in Acute Neuroendocrine Responses to Stressors in Rodents and Humans. Cold Spring Harb. Perspect. Biol..

[25] Hermans E.J., Henckens M.J.A.G., Joëls M., Fernández G. (2014). Dynamic adaptation of large-scale brain networks in response to acute stressors. Trends Neurosci..

[26] Kohn N., Hermans E.J., Fernández G. (2017). Cognitive benefit and cost of acute stress is differentially modulated by individual brain state. Soc. Cogn. Affect. Neurosci..

[27] Kendler K.S., Karkowski-Shuman L. (1997). Stressful life events and genetic liability to major depression: Genetic control of exposure to the environment?. Psychol. Med..

[28] Richter-Levin G., Xu L. (2018). How could stress lead to major depressive disorder?. IBRO Rep..

[29] McManus E., Haroon H., Duncan N.W., Elliott R., Muhlert N. (2022). The effects of stress across the lifespan on the brain, cognition and mental health: A UK biobank study. Neurobiol. Stress.

[30] Charcot J.M. (1879). Lectures on the Diseases of the Nervous System Delivered at La Salpêtrière.

[31] Zou K., Guo W., Tang G., Zheng B., Zheng Z. (2013). A Case of early onset Parkinson’s disease after major stress. Neuropsychiatr. Dis. Treat..

[32] Hemmerle A.M., Herman J.P., Seroogy K.B. (2012). Stress, depression and Parkinson’s disease. Exp. Neurol..

[33] Murphy F., Nasa A., Cullinane D., Raajakesary K., Gazzaz A., Sooknarine V., Haines M., Roman E., Kelly L., O’Neill A. (2022). Childhood Trauma, the HPA Axis and Psychiatric Illnesses: A Targeted Literature Synthesis. Front. Psychiatry.

[34] Inoue T., Tsuchiya K., Koyama T. (1994). Regional changes in dopamine and serotonin activation with various intensity of physical and psychological stress in the rat brain. Pharmacol. Biochem. Behav..

[35] Lupien S.J., McEwen B.S., Gunnar M.R., Heim C. (2009). Effects of stress throughout the lifespan on the brain, behaviour and cognition. Nat. Rev. Neurosci..

[36] He K.-J., Zhang Y.-T., Wei S.-z., Jiang S.-m., Xu L., Ren C., Wang F. (2020). Impact of Maternal Separation on Dopamine System and its Association with Parkinson’s Disease. Neuromol. Med..

[37] Burtscher J., Copin J.C., Rodrigues J., Kumar S.T., Chiki A., Guillot de Suduiraut I., Sandi C., Lashuel H.A. (2019). Chronic corticosterone aggravates behavioral and neuronal symptomatology in a mouse model of alpha-synuclein pathology. Neurobiol. Aging.

[38] Soares N.M., Pereira G.M., Altmann V., de Almeida R.M.M., Rieder C.R.M. (2019). Cortisol levels, motor, cognitive and behavioral symptoms in Parkinson’s disease: A systematic review. J. Neural Transm..

[39] Austin K.W., Ameringer S.W., Cloud L.J. (2016). An Integrated Review of Psychological Stress in Parkinson’s Disease: Biological Mechanisms and Symptom and Health Outcomes. Park. Dis..

[40] Jones M.B., Gates R., Gibson L., Broadway D., Bhatti G., Tea J., Guerra A., Li R., Varman B., Elammari M. (2023). Post-Traumatic Stress Disorder and Risk of Degenerative Synucleinopathies: Systematic Review and Meta-Analysis. Am. J. Geriatr. Psychiatry.

[41] Danan D., Grosskopf Y., Hayut Y., Toledano Y., Doenyas-Barak K., Mayo A., Alon U. (2025). A Mechanistic Model for the HPA Axis Cortisol Paradox in PTSD Dor. bioRxiv.

[42] Juruena M.F., Bocharova M., Agustini B., Young A.H. (2018). Atypical depression and non-atypical depression: Is HPA axis function a biomarker? A systematic review. J. Affect. Disord..

[43] Eo H., Kim S., Jung U.J., Kim S.R. (2024). Alpha-Synuclein and Microglia in Parkinson’s Disease: From Pathogenesis to Therapeutic Prospects. J. Clin. Med..

[44] Nakos Bimpos M., Karali K., Antoniou C., Palermos D., Fouka M., Delis A., Tzieras I., Chrousos G.P., Koutmani Y., Stefanis L. (2024). Alpha-synuclein-induced stress sensitivity renders the Parkinson’s disease brain susceptible to neurodegeneration. Acta Neuropathol. Commun..

[45] Jamiesonm P. (2015). Major Depressive Disorder & Posttraumatic Stress Disorder in Relation to Stress Response Plasticity: An Evolutionary Perspective on Mental Health. Doctoral Dissertation.

[46] Bareeqa S.B., Samar S.S., Kamal S., Masood Y., Allahyar A., Ahmed S.I., Hayat G. (2022). Prodromal depression and subsequent risk of developing Parkinson’s disease: A systematic review with meta-analysis. Neurodegener. Dis. Manag..

[47] Yang J.Z., Kang C.Y., Yuan J., Zhang Y., Wei Y.J., Xu L., Zhou F., Fan X. (2021). Effect of adverse childhood experiences on hypothalamic-pituitary-adrenal (HPA) axis function and antidepressant efficacy in untreated first episode patients with major depressive disorder. Psychoneuroendocrinology.

[48] Chang C.H., Grace A.A. (2014). Amygdala-ventral pallidum pathway decreases dopamine activity after chronic mild stress in rats. Biol. Psychiatry.

[49] Han X., Shao W., Liu Z., Fan S., Yu J., Chen J., Qiao R., Zhou J., Xie P. (2015). iTRAQ-based quantitative analysis of hippocampal postsynaptic density-associated proteins in a rat chronic mild stress model of depression. Neuroscience.

[50] Antoniuk S., Bijata M., Ponimaskin E., Wlodarczyk J. (2019). Chronic unpredictable mild stress for modeling depression in rodents: Meta-analysis of model reliability. Neurosci. Biobehav. Rev..

[51] Ren C., Li L.X., Dong A.Q., Zhang Y.T., Hu H., Mao C.J., Wang F., Liu C.F. (2021). Depression Induced by Chronic Unpredictable Mild Stress Increases Susceptibility to Parkinson’s Disease in Mice via Neuroinflammation Mediated by P2X7 Receptor. ACS Chem. Neurosci..

[52] Janakiraman U., Manivasagam T., Justin Thenmozhi A., Dhanalakshmi C., Essa M.M., Song B.J., Guillemin G.J. (2017). Chronic mild stress augments MPTP induced neurotoxicity in a murine model of Parkinson’s disease. Physiol. Behav..

[53] Xia D., Xiong M., Yang Y., Wang X., Chen Q., Li S., Meng L., Zhang Z. (2025). Chronic stress induces depression-like behaviors and Parkinsonism via upregulating α-synuclein. npj Park. Dis..

[54] Zhang Z., Chu S.F., Wang S.S., Jiang Y.N., Gao Y., Yang P.F., Ai Q.-D., Chen N.H. (2018). RTP801 is a critical factor in the neurodegeneration process of A53T α-synuclein in a mouse model of Parkinson’s disease under chronic restraint stress. Br. J. Pharmacol..

[55] Moylan S., Maes M., Wray N.R., Berk M. (2013). The neuroprogressive nature of major depressive disorder: Pathways to disease evolution and resistance, and therapeutic implications. Mol. Psychiatry.

[56] Liu J., Fan Y., Zeng L.-L., Liu B., Ju Y., Wang M., Dong Q., Lu X., Sun J., Zhang L. (2021). The neuroprogressive nature of major depressive disorder: Evidence from an intrinsic connectome analysis. Transl. Psychiatry.

[57] Boyd R.J., Avramopoulos D., Jantzie L.L., McCallion A.S. (2022). Neuroinflammation represents a common theme amongst genetic and environmental risk factors for Alzheimer and Parkinson diseases. J. Neuroinflammation.

[58] Xu M., Bohlen J.K., Moore C., Nipper M.A., Finn D.A., Jones C.E., Lim M.M., Meshul C.K. (2019). Effects of sleep disruption on stress, nigrostriatal markers, and behavior in a chronic/progressive MPTP male mouse model of parkinsonism. J. Neurosci. Res..

[59] Chan Y.L.E., Bai Y.M., Hsu J.W., Huang K.L., Su T.P., Li C.T., Lin W.C., Pan T.L., Chen T.J., Tsai S.J. (2017). Post-traumatic Stress Disorder and Risk of Parkinson Disease: A Nationwide Longitudinal Study. Am. J. Geriatr. Psychiatry.

[60] Neylan T.C. (2020). Post-traumatic Stress Disorder and Neurodegeneration. Am. J. Geriatr. Psychiatry.

[61] Barer Y., Chodick G., Glaser Chodick N., Gurevich T. (2022). Risk of Parkinson Disease Among Adults With vs Without Posttraumatic Stress Disorder. JAMA Netw. Open.

[62] Miyazaki M., Yagihara H., Fujita H., Yamakado H., Wada K., Minakawa E.N. (2025). Chronic sleep fragmentation accererates the development of prodromal symptoms and pathological changes in Parkinson’s disease model mice. Int. J. Neuropsychopharmacol..

[63] Musiek E.S., Holtzman D.M. (2016). Mechanisms linking circadian clocks, sleep, and neurodegeneration. Science.

[64] van der Heide A., Meinders M.J., Speckens A.E.M., Peerbolte T.F., Bloem B.R., Helmich R.C. (2021). Stress and Mindfulness in Parkinson’s Disease: Clinical Effects and Potential Underlying Mechanisms. Mov. Disord..

[65] van der Heide A., Speckens A.E.M., Meinders M.J., Rosenthal L.S., Bloem B.R., Helmich R.C. (2021). Stress and mindfulness in Parkinson’s disease—A survey in 5000 patients. NPJ Park. Dis..

[66] Wu K.Y., Lin K.J., Chen C.H., Chen C.S., Liu C.Y., Huang S.Y., Yen T.C., Hsiao I.T. (2018). Diversity of neurodegenerative pathophysiology in nondemented patients with major depressive disorder: Evidence of cerebral amyloidosis and hippocampal atrophy. Brain Behav..

[67] Kline S.A., Mega M.S. (2020). Stress-Induced Neurodegeneration: The Potential for Coping as Neuroprotective Therapy. Am. J. Alzheimer’s Dis. Other Dement..

[68] Wirth M., Lange C., Huijbers W. (2019). Plasma cortisol is associated with cerebral hypometabolism across the Alzheimer’s disease spectrum. Neurobiol. Aging.

[69] Sic A., Cvetkovic K., Manchanda E., Knezevic N.N. (2024). Neurobiological Implications of Chronic Stress and Metabolic Dysregulation in Inflammatory Bowel Diseases. Diseases.

[70] Claros S., Gil A., Martinelli M., Valverde N., Lara E., Boraldi F., Pavia J., Martín-Montañez E., Garcia-Fernandez M. (2021). Impact of Glucocorticoid on a Cellular Model of Parkinson’s Disease: Oxidative Stress and Mitochondrial Function. Brain Sci..

[71] Ibrahimagic O.C., Jakubovic A.C., Smajlovic D., Dostovic Z., Kunic S., Iljazovic A. (2016). Psychological Stress and Changes of Hypothalamic-Pituitary-Adrenal Axis in Patients with “De Novo” Parkinson’s Disease. Med. Arch..

[72] Aggarwal N.T., Wilson R.S., Beck T.L., Rajan K.B., Mendes De Leon C.F., Evans D.A., Everson-Rose S.A. (2014). Perceived stress and change in cognitive function among adults 65 years and older. Psychosom. Med..

[73] Peavy G.M., Salmon D.P., Jacobson M.W., Hervey A., Gamst A.C., Wolfson T., Patterson T.L., Goldman S., Mills P.J., Khandrika S. (2009). Effects of chronic stress on memory decline in cognitively normal and mildly impaired older adults. Am. J. Psychiatry.

[74] Hellhammer D.H., Wüst S., Kudielka B.M. (2009). Salivary cortisol as a biomarker in stress research. Psychoneuroendocrinology.

[75] Mayer S.E., Snodgrass M., Liberzon I., Briggs H., Curtis G.C., Abelson J.L. (2017). The psychology of HPA axis activation: Examining subjective emotional distress and control in a phobic fear exposure model. Psychoneuroendocrinology.

[76] Riachi E., Holma J., Laitila A. (2024). Psychotherapists’ perspectives on loss of sense of control. Brain Behav..

[77] Molina P., Andero R., Armario A. (2023). Restraint or immobilization: A comparison of methodologies for restricting free movement in rodents and their potential impact on physiology and behavior. Neurosci. Biobehav. Rev..

[78] Armario A. (2021). The forced swim test: Historical, conceptual and methodological considerations and its relationship with individual behavioral traits. Neurosci. Biobehav. Rev..

[79] Markov D.D., Novosadova E.V. (2022). Chronic Unpredictable Mild Stress Model of Depression: Possible Sources of Poor Reproducibility and Latent Variables. Biology.

[80] Malta M.B., Martins J., Novaes L.S., dos Santos N.B., Sita L., Camarini R., Scavone C., Bittencourt J., Munhoz C.D. (2021). Norepinephrine and Glucocorticoids Modulate Chronic Unpredictable Stress-Induced Increase in the Type 2 CRF and Glucocorticoid Receptors in Brain Structures Related to the HPA Axis Activation. Mol. Neurobiol..

[81] Demuyser T., Bentea E., Deneyer L., Albertini G., Massie A., Smolders I. (2016). Disruption of the HPA-axis through corticosterone-release pellets induces robust depressive-like behavior and reduced BDNF levels in mice. Neurosci. Lett..

[82] Kositsyn Y.M.H.B., Volgin A.D., de Abreu M.S., Demin K.A., Zabegalov K.N., Maslov G.O., Petersen E.V., Kolesnikova T.O., Strekalova T., Kalueff A.V. (2022). Towards translational modeling of behavioral despair and its treatment in zebrafish. Behav. Brain Res..

[83] Li X.-H., Liu N.-B., Zhang M.-H., Zhou Y.-L., Liao J.-W., Liu X.-Q., Chen H.-W. (2007). Effects of chronic multiple stress on learning and memory and the expression of Fyn, BDNF, TrkB in the hippocampus of rats. Chin. Med. J..

[84] Liu R.T., Kleiman E.M., Nestor B.A., Cheek S.M. (2015). The Hopelessness Theory of Depression: A Quarter Century in Review. Clin. Psychol..

[85] Du X., Pang T.Y. (2015). Is Dysregulation of the HPA-Axis a Core Pathophysiology Mediating Co-Morbid Depression in Neurodegenerative Diseases?. Front. Psychiatry.

[86] Stetler C., Miller G.E. (2011). Depression and hypothalamic-pituitary-adrenal activation: A quantitative summary of four decades of research. Psychosom. Med..

[87] Sahu M., Dubey R., Chandrakar A., Kumar M., Kumar M. (2022). A systematic review and meta-analysis of serum and plasma cortisol levels in depressed patients versus control. Indian J. Psychiatry.

[88] Jopling E., LeMoult J. (2023). Childhood adversity and the cortisol awakening response in depression: A meta-analysis. J. Mood Anxiety Disord..

[89] Bielajew C., Konkle A.T.M., Merali Z. (2002). The effects of chronic mild stress on male Sprague-Dawley and Long Evans rats: I. Biochemical and physiological analyses. Behav. Brain Res..

[90] de Andrade J.S., Céspedes I.C., Abrão R.O., dos Santos T.B., Diniz L., Britto L.R.G., Spadari-Bratfisch R.C., Ortolani D., Melo-Thomas L., da Silva R.C.B. (2013). Chronic unpredictable mild stress alters an anxiety-related defensive response, Fos immunoreactivity and hippocampal adult neurogenesis. Behav. Brain Res..

[91] Horstmann S., Dose T., Lucae S., Kloiber S., Menke A., Hennings J., Spieler D., Uhr M., Holsboer F., Ising M. (2009). Suppressive effect of mirtazapine on the HPA system in acutely depressed women seems to be transient and not related to antidepressant action. Psychoneuroendocrinology.

[92] Raison C.L., Miller A.H. (2003). When not enough is too much: The role of insufficient glucocorticoid signaling in the pathophysiology of stress-related disorders. Am. J. Psychiatry.

[93] Young E.A., Lopez J.F., Murphy-Weinberg V., Watson S.J., Akil H. (2003). Mineralocorticoid receptor function in major depression. Arch. Gen. Psychiatry.

[94] Ke Q., Li R., Cai L., Wu S.-D., Li C.-M. (2019). Ro41-5253, a selective antagonist of retinoic acid receptor α ameliorates chronic unpredictable mild stress-induced depressive-like behaviors in rats: Involvement of regulating HPA axis and improving hippocampal neuronal deficits. Brain Res. Bull..

[95] Patel P.D., Lopez J.F., Lyons D.M., Burke S., Wallace M., Schatzberg A.F. (2000). Glucocorticoid and mineralocorticoid receptor mRNA expression in squirrel monkey brain. J. Psychiatr. Res..

[96] Abercrombie H.C., Jahn A.L., Davidson R.J., Kern S., Kirschbaum C., Halverson J. (2011). Cortisol’s effects on hippocampal activation in depressed patients are related to alterations in memory formation. J. Psychiatr. Res..

[97] Nooshinfar E., Akbarzadeh-Baghban A., Meisami E. (2011). Effects of increasing durations of immobilization stress on plasma corticosterone level, learning and memory and hippocampal BDNF gene expression in rats. Neurosci. Lett..

[98] Sapolsky R.M. (2000). The possibility of neurotoxicity in the hippocampus in major depression: A primer on neuron death. Biol. Psychiatry.

[99] Lee A.L., Ogle W.O., Sapolsky R.M. (2002). Stress and depression: Possible links to neuron death in the hippocampus. Bipolar Disord..

[100] Spiers J.G., Chen H.J.C., Sernia C., Lavidis N.A. (2015). Activation of the hypothalamic-pituitary-adrenal stress axis induces cellular oxidative stress. Front. Neurosci..

[101] Tata D.A., Marciano V.A., Anderson B.J. (2006). Synapse loss from chronically elevated glucocorticoids: Relationship to neuropil volume and cell number in hippocampal area CA3. J. Comp. Neurol..

[102] Moica T., Gligor A., Moica S. (2016). The Relationship between Cortisol and the Hippocampal Volume in Depressed Patients–A MRI Pilot Study. Procedia Technol..

[103] Hickie I., Naismith S., Ward P.B., Turner K., Scott E., Mitchell P., Wilhelm K., Parker G. (2005). Reduced hippocampal volumes and memory loss in patients with early- and late-onset depression. Br. J. Psychiatry.

[104] Woon F.L., Sood S., Hedges D.W. (2010). Hippocampal volume deficits associated with exposure to psychological trauma and posttraumatic stress disorder in adults: A meta-analysis. Prog. Neuro-Psychopharmacol. Biol. Psychiatry.

[105] Luo Y., Cao Z., Wang D., Wu L., Li Y., Sun W., Zhu Y. (2014). Dynamic study of the hippocampal volume by structural MRI in a rat model of depression. Neurol. Sci..

[106] Li Y., Zhu X., Ju S., Yan J., Wang D., Zhu Y., Zang F. (2017). Detection of volume alterations in hippocampal subfields of rats under chronic unpredictable mild stress using 7T MRI: A follow-up study. J. Magn. Reson. Imaging.

[107] Alqurashi G.K., Hindi E.A., Zayed M.A., Abd El-Aziz G.S., Alturkistani H.A., Ibrahim R.F., Al-Thepyani M.A., Bakhlgi R., Alzahrani N.A., Ashraf G.M. (2022). The Impact of Chronic Unpredictable Mild Stress-Induced Depression on Spatial, Recognition and Reference Memory Tasks in Mice: Behavioral and Histological Study. Behav. Sci..

[108] Zahedi E., Kavyannejad R., Salami P., Sadr S.S. (2025). Modulating depressive-like behaviors, memory impairment, and oxidative stress in chronic stress rat model using visible light therapy. Sci. Rep..

[109] Gilbertson M.W., Shenton M.E., Ciszewski A., Kasai K., Lasko N.B., Orr S.P., Pitman R.K. (2002). Smaller hippocampal volume predicts pathologic vulnerability to psychological trauma. Nat. Neurosci..

[110] Sapolsky R.M., Krey L.C., McEwen B.S. (1986). The neuroendocrinology of stress and aging: The glucocorticoid cascade hypothesis. Endocr. Rev..

[111] Zhe D., Fang H., Yuxiu S. (2008). Expressions of hippocampal mineralocorticoid receptor (MR) and glucocorticoid receptor (GR) in the single-prolonged stress-rats. Acta Histochem. Cytochem..

[112] Villar-Conde S., Astillero-Lopez V., Gonzalez-Rodriguez M., Villanueva-Anguita P., Saiz-Sanchez D., Martinez-Marcos A., Flores-Cuadrado A., Ubeda-Bañon I. (2021). The Human Hippocampus in Parkinson’s Disease: An Integrative Stereological and Proteomic Study. J. Park. Dis..

[113] Bao A.M., Swaab D.F. (2019). The human hypothalamus in mood disorders: The HPA axis in the center. IBRO Rep..

[114] Wadsworth M.E., Broderick A.V., Loughlin-Presnal J.E., Bendezu J.J., Joos C.M., Ahlkvist J.A., Perzow S.E.D., McDonald A. (2019). Co-activation of SAM and HPA Responses to Acute Stress: A Review of the Literature and Test of Differential Associations with Preadolescents’ Internalizing and Externalizing. Dev. Psychobiol..

[115] Kaiser M., Jaillardon L. (2023). Pathogenesis of the crosstalk between reproductive function and stress in animals-part 1: Hypothalamo-pituitary-adrenal axis, sympatho-adrenomedullary system and kisspeptin. Reprod. Domest. Anim..

[116] Rommelfanger K.S., Weinshenker D. (2007). Norepinephrine: The redheaded stepchild of Parkinson’s disease. Biochem. Pharmacol..

[117] Hamon M., Blier P. (2013). Monoamine neurocircuitry in depression and strategies for new treatments. Prog. Neuro-Psychopharmacol. Biol. Psychiatry.

[118] Foote S.L., Bloom F.E., Aston Jones G. (1983). Nucleus locus ceruleus: New evidence of anatomical and physiological specificity. Physiol. Rev..

[119] Zarow C., Lyness S.A., Mortimer J.A., Chui H.C. (2003). Neuronal loss is greater in the locus coeruleus than nucleus basalis and substantia nigra in Alzheimer and Parkinson diseases. Arch. Neurol..

[120] Fornai F., di Poggio A., Pellegrini A., Ruggieri S., Paparelli A. (2007). Noradrenaline in Parkinson’s disease: From disease progression to current therapeutics. Curr. Med. Chem..

[121] McMillan P.J., White S.S., Franklin A., Greenup J.L., Leverenz J.B., Raskind M.A., Szot P. (2011). Differential response of the central noradrenergic nervous system to the loss of locus coeruleus neurons in Parkinson’s disease and Alzheimer’s disease. Brain Res..

[122] Zhou C., Guo T., Bai X., Wu J.J., Gao T., Guan X., Liu X., Gu L., Huang P., Xuan M. (2021). Locus coeruleus degeneration is associated with disorganized functional topology in Parkinson’s disease. NeuroImage Clin..

[123] Rommelfanger K.S., Edwards G.L., Freeman K.G., Liles L.C., Miller G.W., Weinshenker D. (2007). Norepinephrine loss produces more profound motor deficits than MPTP treatment in mice. Proc. Natl. Acad. Sci. USA.

[124] El Mansari M., Guiard B.P., Chernoloz O., Ghanbari R., Katz N., Blier P. (2010). Relevance of norepinephrine-dopamine interactions in the treatment of major depressive disorder. CNS Neurosci. Ther..

[125] Holly E.N., Miczek K.A. (2015). Ventral tegmental area dopamine revisited: Effects of acute and repeated stress. Psychopharmacology.

[126] Park J.W., Bhimani R.V., Park J. (2017). Noradrenergic Modulation of Dopamine Transmission Evoked by Electrical Stimulation of the Locus Coeruleus in the Rat Brain. ACS Chem. Neurosci..

[127] Rosin D. (1996). Distribution of alpha 2C-adrenergic receptor-like immunoreactivity in the rat central nervous system. J. Comp. Neurol..

[128] Zhang W., Ordway G.A. (2003). The α2C-adrenoceptor modulates GABA release in mouse striatum. Mol. Brain Res..

[129] Alachkar A., Brotchie J., Jones O.T. (2006). α2-Adrenoceptor-mediated modulation of the release of GABA and noradrenaline in the rat substantia nigra pars reticulata. Neurosci. Lett..

[130] Inyushin M.U., Arencibia-Albite F., Vázquez-Torres R., Vélez-Hernández M.E., Jiménez-Rivera C.A. (2010). Alpha-2 noradrenergic receptor activation inhibits the hyperpolarization-activated cation current (Ih) in neurons of the ventral tegmental area. Neuroscience.

[131] Cools A.R., Tuinstra T. (2002). Neurochemical Evidence that Mesolimbic Noradrenaline Directs Mesolimbic Dopamine, Implying that Noradrenaline, Like Dopamine, Plays a Key Role in Goal-Directed and Motivational Behavior. The Basal Ganglia VI.

[132] Schroeter S., Apparsundaram S., Wiley R.G., Miner L.H., Sesack S.R., Blakely R.D. (2010). Immunolocalization of the cocaine- and antidepressant-sensitive l-norepinephrine transporter. J. Comp. Neurol..

[133] Kilbourn M.R., Sherman P.S., Abbott L.C. (1998). Reduced MPTP neurotoxicity in striatum of the mutant mouse tottering. Synapse.

[134] Rommelfanger K.S., Weinshenker D., Miller G.W. (2004). Reduced MPTP toxicity in noradrenaline transporter knockout mice. J. Neurochem..

[135] Chen M.J., Nguyen T.V., Pike C.J., Russo-Neustadt A.A. (2007). Norepinephrine induces BDNF and activates the PI-3K and MAPK cascades in embryonic hippocampal neurons. Cell. Signal..

[136] Li X.L., Liu H., Liu S.H., Cheng Y., Xie G.J. (2022). Intranasal Administration of Brain-Derived Neurotrophic Factor Rescues Depressive-Like Phenotypes in Chronic Unpredictable Mild Stress Mice. Neuropsychiatr. Dis. Treat..

[137] Feinstein D.L., Kalinin S., Braun D. (2016). Causes, consequences, and cures for neuroinflammation mediated via the locus coeruleus: Noradrenergic signaling system. J. Neurochem..

[138] Ross J.A., Van Bockstaele E.J. (2021). The Locus Coeruleus-Norepinephrine System in Stress and Arousal: Unraveling Historical, Current, and Future Perspectives. Front. Psychiatry.

[139] Wang B., Wang Y., Wu Q., Huang H.P., Li S. (2017). Effects of α2A Adrenoceptors on Norepinephrine Secretion from the Locus Coeruleus during Chronic Stress-Induced Depression. Front. Neurosci..

[140] Martín-Hernández D., Pereira M.P., Tendilla-Beltrán H., Madrigal J.L.M., García-Bueno B., Leza J.C., Caso J.R. (2019). Modulation of Monoaminergic Systems by Antidepressants in the Frontal Cortex of Rats After Chronic Mild Stress Exposure. Mol. Neurobiol..

[141] Chen P., Fan Y., Li Y., Sun Z., Bissette G., Zhu M.Y. (2012). Chronic social defeat up-regulates expression of norepinephrine transporter in rat brains. Neurochem. Int..

[142] Seki K., Yoshida S., Jaiswal M. (2018). Molecular mechanism of noradrenaline during the stress-induced major depressive disorder. Neural Regen. Res..

[143] Klimek V., Stockmeier C., Overholser J., Meltzer H.Y., Kalka S., Dilley G., Ordway G.A. (1997). Reduced levels of norepinephrine transporters in the locus coeruleus in major depression. J. Neurosci..

[144] Gómez Esteban J.C., Azkue N., Acera M.Á., Tijero B., Gabilondo I., Sanchez Pernaute M.R. (2026). Locus coeruleus: Clinical aspects of its involvement in Parkinson’s disease. Neurol. Perspect..

[145] Paredes-Rodriguez E., Vegas-Suarez S., Morera-Herreras T., De Deurwaerdere P., Miguelez C. (2020). The Noradrenergic System in Parkinson’s Disease. Front. Pharmacol..

[146] Van Der Heide A., Trenkwalder C., Bloem B.R., Helmich R.C. (2024). The Last Straw: How Stress Can Unmask Parkinson’s Disease. J. Park. Dis..

[147] Snyder A.M., Stricker E.M., Zigmond M.J. (1985). Stress-induced neurological impairments in an animal model of parkinsonism. Ann. Neurol..

[148] Biswas K., Alexander K., Francis M.M. (2022). Reactive Oxygen Species: Angels and Demons in the Life of a Neuron. NeuroSci.

[149] Ebding J., Mazzone F., Kins S., Pielage J., Maritzen T. (2025). How neurons cope with oxidative stress. Biol. Chem..

[150] Hassan W., Noreen H., Rehman S., Kamal M.A., da Rocha J.B.T. (2022). Association of Oxidative Stress with Neurological Disorders. Curr. Neuropharmacol..

[151] Brazdis R.M., Alecu J.E., Marsch D., Dahms A., Simmnacher K., Lörentz S., Brendler A., Schneider Y., Marxreiter F., Roybon L. (2020). Demonstration of brain region-specific neuronal vulnerability in human iPSC-based model of familial Parkinson’s disease. Hum. Mol. Genet..

[152] Zampese E., Surmeier D.J. (2020). Calcium, Bioenergetics, and Parkinson’s Disease. Cells.

[153] Surmeier D.J. (2017). Determinants of dopaminergic neuron loss in Parkinson’s disease. FEBS J..

[154] Herbet M., Korga A., Gawrońska-Grzywacz M., Izdebska M., Piątkowska-Chmiel I., Poleszak E., Wróbel A., Matysiak W., Jodłowska-Jȩdrych B., Dudka J. (2017). Chronic Variable Stress Is Responsible for Lipid and DNA Oxidative Disorders and Activation of Oxidative Stress Response Genes in the Brain of Rats. Oxidative Med. Cell. Longev..

[155] Şimşek Ş., Yüksel T., Kaplan İ., Uysal C., Aktaş H. (2016). The Levels of Cortisol and Oxidative Stress and DNA Damage in Child and Adolescent Victims of Sexual Abuse with or without Post-Traumatic Stress Disorder. Psychiatry Investig..

[156] Salim S. (2014). Oxidative stress and psychological disorders. Curr. Neuropharmacol..

[157] Bhatt S., Nagappa A.N., Patil C.R. (2020). Role of oxidative stress in depression. Drug Discov. Today.

[158] Maes M., Galecki P., Chang Y.S., Berk M. (2011). A review on the oxidative and nitrosative stress (O&NS) pathways in major depression and their possible contribution to the (neuro)degenerative processes in that illness. Prog. Neuro-Psychopharmacol. Biol. Psychiatry.

[159] Chandley M.J., Szebeni A., Szebeni K., Wang-Heaton H., Garst J., Stockmeier C.A., Lewis N.H., Ordway G.A. (2022). Markers of elevated oxidative stress in oligodendrocytes captured from the brainstem and occipital cortex in major depressive disorder and suicide. Prog. Neuropsychopharmacol. Biol. Psychiatry.

[160] Connor J.R., Menzies S.L., Martin S.M.S., Mufson E.J. (1990). Cellular distribution of transferrin, ferritin, and iron in normal and aged human brains. J. Neurosci. Res..

[161] Badaracco M.E., Siri M.V.R., Pasquini J.M. (2010). Oligodendrogenesis: The role of iron. Biofactors.

[162] Thomas G.E.C., Zarkali A., Ryten M., Shmueli K., Gil-Martinez A.L., Leyland L.A., McColgan P., Acosta-Cabronero J., Lees A.J., Weil R.S. (2021). Regional brain iron and gene expression provide insights into neurodegeneration in Parkinson’s disease. Brain.

[163] Han J., Fan Y., Wu P., Huang Z., Li X., Zhao L., Ji Y., Zhu M. (2021). Parkinson’s Disease Dementia: Synergistic Effects of Alpha-Synuclein, Tau, Beta-Amyloid, and Iron. Front. Aging Neurosci..

[164] Simpson D.S.A., Oliver P.L. (2020). ROS Generation in Microglia: Understanding Oxidative Stress and Inflammation in Neurodegenerative Disease. Antioxidants.

[165] Beckhauser T.F., Francis-Oliveira J., De Pasquale R. (2016). Reactive Oxygen Species: Physiological and Physiopathological Effects on Synaptic Plasticity. J. Exp. Neurosci..

[166] Cobley J.N., Fiorello M.L., Bailey D.M. (2018). 13 reasons why the brain is susceptible to oxidative stress. Redox Biol..

[167] Crichton R. (2002). Inorganic Biochemistry of Iron Metabolism: From Molecular Mechanisms to Clinical Consequences.

[168] Thirupathi A., Chang Y.Z. (2019). Brain Iron Metabolism and CNS Diseases. Adv. Exp. Med. Biol..

[169] Beard J. (2003). Iron deficiency alters brain development and functioning. J. Nutr..

[170] Burhans M.S., Dailey C., Beard Z., Wiesinger J., Murray-Kolb L., Jones B.C., Beard J.L. (2005). Iron deficiency: Differential effects on monoamine transporters. Nutr. Neurosci..

[171] Wilms H., Rosenstiel P., Sievers J., Deuschl G., Zecca L., Lucius R. (2003). Activation of microglia by human neuromelanin is NF-kappaB dependent and involves p38 mitogen-activated protein kinase: Implications for Parkinson’s disease. FASEB J..

[172] Zucca F.A., Giaveri G., Gallorini M., Albertini A., Toscani M., Pezzoli G., Lucius R., Wilms H., Sulzer D., Ito S. (2004). The neuromelanin of human substantia nigra: Physiological and pathogenic aspects. Pigment Cell Res..

[173] Viceconte N., Burguillos M.A., Herrera A.J., De Pablos R.M., Joseph B., Venero J.L. (2015). Neuromelanin activates proinflammatory microglia through a caspase-8-dependent mechanism. J. Neuroinflammation.

[174] Kirsch M., Korth H.G., Stenert V., Sustmann R., De Groot H. (2003). The autoxidation of tetrahydrobiopterin revisited. Proof of superoxide formation from reaction of tetrahydrobiopterin with molecular oxygen. J. Biol. Chem..

[175] Kim S.T., Choi J.H., Chang J.W., Kim S.W., Hwang O. (2005). Immobilization stress causes increases in tetrahydrobiopterin, dopamine, and neuromelanin and oxidative damage in the nigrostriatal system. J. Neurochem..

[176] Sulzer D., Bogulavsky J., Larsen K.E., Behr G., Karatekin E., Kleinman M.H., Turro N., Krantz D., Edwards R.H., Greene L.A. (2000). Neuromelanin biosynthesis is driven by excess cytosolic catecholamines not accumulated by synaptic vesicles. Proc. Natl. Acad. Sci. USA.

[177] Liang C.L., Nelson O., Yazdani U., Pasbakhsh P., German D.C. (2004). Inverse relationship between the contents of neuromelanin pigment and the vesicular monoamine transporter-2: Human midbrain dopamine neurons. J. Comp. Neurol..

[178] Zucca F.A., Segura-Aguilar J., Ferrari E., Muñoz P., Paris I., Sulzer D., Sarna T., Casella L., Zecca L. (2017). Interactions of iron, dopamine and neuromelanin pathways in brain aging and Parkinson’s disease. Prog. Neurobiol..

[179] Wulf M., Barkovits K., Schork K., Eisenacher M., Riederer P., Gerlach M., Eggers B., Marcus K. (2022). Neuromelanin granules of the substantia nigra: Proteomic profile provides links to tyrosine hydroxylase, stress granules and lysosomes. J. Neural Transm..

[180] Zecca L., Zucca F.A., Wilms H., Sulzer D. (2003). Neuromelanin of the substantia nigra: A neuronal black hole with protective and toxic characteristics. Trends Neurosci..

[181] Wang M., Wang H., Wang J., Lu S., Li C., Zhong X., Wang N., Ge R., Zheng Q., Chen J. (2022). Modified Iron Deposition in Nigrosomes by Pharmacotherapy for the Management of Parkinson’s Disease. Front. Mol. Biosci..

[182] Costas C., Faro L.R.F. (2022). Do Naturally Occurring Antioxidants Protect Against Neurodegeneration of the Dopaminergic System? A Systematic Revision in Animal Models of Parkinson’s Disease. Curr. Neuropharmacol..

[183] Mittal P., Dhankhar S., Chauhan S., Garg N., Bhattacharya T., Ali M., Chaudhary A.A., Rudayni H.A., Al-Zharani M., Ahmad W. (2023). A Review on Natural Antioxidants for Their Role in the Treatment of Parkinson’s Disease. Pharmaceuticals.

[184] Duarte-Jurado A.P., Gopar-Cuevas Y., Saucedo-Cardenas O., Loera-Arias M.d.J., Montes-De-oca-luna R., Garcia-Garcia A., Rodriguez-Rocha H. (2021). Antioxidant Therapeutics in Parkinson’s Disease: Current Challenges and Opportunities. Antioxidants.

[185] Pacholko A.G., Wotton C.A., Bekar L.K. (2020). Astrocytes-The Ultimate Effectors of Long-Range Neuromodulatory Networks?. Front. Cell. Neurosci..

[186] Serapide M.F., L’Episcopo F., Tirolo C., Testa N., Caniglia S., Giachino C., Marchetti B. (2020). Boosting Antioxidant Self-defenses by Grafting Astrocytes Rejuvenates the Aged Microenvironment and Mitigates Nigrostriatal Toxicity in Parkinsonian Brain via an Nrf2-Driven Wnt/β-Catenin Prosurvival Axis. Front. Aging Neurosci..

[187] Grammatopoulos T.N., Jones S.M., Ahmadi F.A., Hoover B.R., Snell L.D., Skoch J., Jhaveri V.V., Poczobutt A.M., Weyhenmeyer J.A., Zawada W.M. (2007). Angiotensin type 1 receptor antagonist losartan, reduces MPTP-induced degeneration of dopaminergic neurons in substantia nigra. Mol. Neurodegener.

[188] Sathiya S., Ranju V., Kalaivani P., Priya R.J., Sumathy H., Sunil A.G., Babu C.S. (2013). Telmisartan attenuates MPTP induced dopaminergic degeneration and motor dysfunction through regulation of α-synuclein and neurotrophic factors (BDNF and GDNF) expression in C57BL/6J mice. Neuropharmacology.

[189] Tsytsarev V., Vaganova A.N., Volnova A., Fesenko Z., Getachew B., Gainetdinov R.R., Tizabi Y. (2025). Role of Glial Trace Amine Associated Receptor 1 (TAAR1) and Microbiota in Schizophrenia. Neurochem. Res..

[190] Abdalkader M., Lampinen R., Kanninen K.M., Malm T.M., Liddell J.R. (2018). Targeting Nrf2 to Suppress Ferroptosis and Mitochondrial Dysfunction in Neurodegeneration. Front. Neurosci..

[191] Suzen S., Tucci P., Profumo E., Buttari B., Saso L. (2022). A Pivotal Role of Nrf2 in Neurodegenerative Disorders: A New Way for Therapeutic Strategies. Pharmaceuticals.

[192] Chen K., Wang H., Ilyas I., Mahmood A., Hou L. (2023). Microglia and Astrocytes Dysfunction and Key Neuroinflammation-Based Biomarkers in Parkinson’s Disease. Brain Sci..

[193] Gcwensa N.Z., Russell D.L., Cowell R.M., Volpicelli-Daley L.A. (2021). Molecular Mechanisms Underlying Synaptic and Axon Degeneration in Parkinson’s Disease. Front. Cell. Neurosci..

[194] Bagnoli E., Diviney T., FitzGerald U. (2021). Dysregulation of astrocytic mitochondrial function following exposure to a dopamine metabolite: Implications for Parkinson’s disease. Eur. J. Neurosci..

[195] Bouvier D.S., Fixemer S., Heurtaux T., Jeannelle F., Frauenknecht K.B.M., Mittelbronn M. (2022). The Multifaceted Neurotoxicity of Astrocytes in Ageing and Age-Related Neurodegenerative Diseases: A Translational Perspective. Front. Physiol..

[196] Stevenson R., Samokhina E., Rossetti I., Morley J.W., Buskila Y. (2020). Neuromodulation of Glial Function During Neurodegeneration. Front. Cell. Neurosci..

[197] Catale C., Carola V., Viscomi M.T. (2022). Early life stress-induced neuroinflammation and neurological disorders: A novel perspective for research. Neural Regen. Res..

[198] Herrera A.J., Espinosa-Oliva A.M., Carrillo-Jiménez A., Oliva-Martín M.J., García-Revilla J., García-Quintanilla A., de Pablos R.M., Venero J.L. (2015). Relevance of chronic stress and the two faces of microglia in Parkinson’s disease. Front. Cell. Neurosci..

[199] van den Heuvel L.L., du Plessis S., Stalder T., Acker D., Kirschbaum C., Carr J., Seedat S. (2020). Hair glucocorticoid levels in Parkinson’s disease. Psychoneuroendocrinology.

[200] De Pablos R.M., Herrera A.J., Espinosa-Oliva A.M., Sarmiento M., Muñoz M.F., Machado A., Venero J.L. (2014). Chronic stress enhances microglia activation and exacerbates death of nigral dopaminergic neurons under conditions of inflammation. J. Neuroinflammation.

[201] Wood S.K. (2020). The role of inflammation and oxidative stress in depression and cardiovascular disease. Cardiovascular Implications of Stress and Depression.

[202] Mosiołek A., Pięta A., Jakima S., Zborowska N., Mosiołek J., Szulc A. (2021). Effects of Antidepressant Treatment on Peripheral Biomarkers in Patients with Major Depressive Disorder (MDD). J. Clin. Med..

[203] Rodríguez De Lores Arnaiz G. (2007). Na^+^, K^+^-ATPase in the Brain: Structure and Function. Handbook of Neurochemistry and Molecular Neurobiology: Neural Membranes and Transport.

[204] Holm T.H., Lykke-Hartmann K. (2016). Insights into the pathology of the α3 Na^+^/K^+^-ATPase ion pump in neurological disorders; lessons from animal models. Front. Physiol..

[205] De Vasconcellos A.P.S., Zugno A.I., Dos Santos A.H.D.P., Nietto F.B., Crema L.M., Gonçalves M., Franzon R., De Souza Wyse A.T., Da Rocha E.R., Dalmaz C. (2005). Na^+^,K^+^-ATPase activity is reduced in hippocampus of rats submitted to an experimental model of depression: Effect of chronic lithium treatment and possible involvement in learning deficits. Neurobiol. Learn. Mem..

[206] Crema L., Schlabitz M., Tagliari B., Cunha A., Simão F., Krolow R., Pettenuzzo L., Salbego C., Vendite D., Wyse A.T.S. (2010). Na^+^, K^+^ ATPase activity is reduced in amygdala of rats with chronic stress-induced anxiety-like behavior. Neurochem. Res..

[207] Streck E.L., Zugno A.I., Tagliari B., Franzon R., Wannmacher C.M.D., Wajner M., Wyse A.T.S. (2001). Inhibition of rat brain Na^+^, K^+^-ATPase activity induced by homocysteine is probably mediated by oxidative stress. Neurochem. Res..

[208] Lima F.D., Souza M.A., Furian A.F., Rambo L.M., Ribeiro L.R., Martignoni F.V., Hoffmann M.S., Fighera M.R., Royes L.F.F., Oliveira M.S. (2008). Na^+^,K^+^-ATPase activity impairment after experimental traumatic brain injury: Relationship to spatial learning deficits and oxidative stress. Behav. Brain Res..

[209] Zhang X., Lee W., Bian J.S. (2022). Recent Advances in the Study of Na^+^/K^+^-ATPase in Neurodegenerative Diseases. Cells.

[210] de Lores Arnaiz G.R., Ordieres M.G.L. (2014). Brain Na^+^, K^+^-ATPase activity in aging and disease. Int. J. Biomed. Sci..

[211] Kirshenbaum G.S., Saltzman K., Rose† B., Petersen J., Vilsen B., Roder J.C. (2011). Decreased neuronal Na^+^,K^+^-ATPase activity in Atp1a3 heterozygous mice increases susceptibility to depression-like endophenotypes by chronic variable stress G. Genes Brain Behav..

[212] Klein L.E., Bartolomei M.S., Lo C.S. (1984). Corticosterone and triiodothyronine control of myocardial Na^+^-K^+^-ATPase activity in rats. Am. J. Physiol..

[213] Wang Z.M., Celsi G. (1993). Glucocorticoids differentially regulate the mRNA for Na^+^, K^+^-ATPase isoforms in infant rat heart. Pediatr. Res..

[214] Golden G.A., Mason R.P., Tulenko T.N., Zubenko G.S., Rubin R.T. (1999). Rapid and opposite effects of cortisol and estradiol on human erythrocyte Na^+^,K^+^-atpase activity: Relationship to steroid intercalation into the cell membrane. Life Sci..

[215] Oselkin M., Tian D., Bergold P.J. (2010). Low-dose cardiotonic steroids increase sodium-potassium ATPase activity that protects hippocampal slice cultures from experimental ischemia. Neurosci. Lett..

[216] Orellana A.M., Kinoshita P.F., Leite J.A., Kawamoto E.M., Scavone C. (2016). Cardiotonic Steroids as Modulators of Neuroinflammation. Front. Endocrinol..

[217] Ferreira D.A., Medeiros A.B.A., Soares M.M., Lima É.d.A., Oliveira G.C.S.L.d., Leite M.B.d.S., Machado M.V., Villar J.A.F.P., Barbosa L.A., Scavone C. (2024). Evaluation of Anti-Inflammatory Activity of the New Cardiotonic Steroid γ-Benzylidene Digoxin 8 (BD-8) in Mice. Cells.

[218] Petrushanko I.Y., Lisitskii D.R., Strelkova M.A., Tolstova A.P., Fedulov A.P., Filonov F.A., Mitkevich V.A., Makarov A.A. (2025). Impact of the diverse cardiotonic steroids on beta-amyloid precursor protein level. Front. Pharmacol..

[219] Bagrov A.Y., Shapiro J.I., Fedorova O.V. (2009). Endogenous cardiotonic steroids: Physiology, pharmacology, and novel therapeutic targets. Pharmacol. Rev..

[220] Fabbri M., Ferreira J.J., Rascol O. (2022). COMT Inhibitors in the Management of Parkinson’s Disease. CNS Drugs.

[221] Napolitano A., Zürcher G., Da Prada M. (1995). Effects of tolcapone, a novel catechol-O-methyltransferase inhibitor, on striatal metabolism of L-DOPA and dopamine in rats. Eur. J. Pharmacol..

[222] Gainetdinov R.R., Hoener M.C., Berry M.D. (2018). Trace amines and their receptors. Pharmacol. Rev..

[223] Berry M.D., Gainetdinov R.R., Hoener M.C., Shahid M. (2017). Pharmacology of human trace amine-associated receptors: Therapeutic opportunities and challenges. Pharmacol. Ther..

[224] Vaganova A.N., Fesenko Z.S., Efimova E.V., Chekrygin S.A., Shafranskaya D.D., Prjibelski A.D., Katolikova N.V., Gainetdinov R.R. (2024). Knocking Out TAAR5: A Pathway to Enhanced Neurogenesis and Dopamine Signaling in the Striatum. Cells.

[225] Sotnikova T.D., Beaulieu J.M., Espinoza S., Masri B., Zhang X., Salahpour A., Barak L.S., Caron M.G., Gainetdinov R.R. (2010). The dopamine metabolite 3-methoxytyramine is a neuromodulator. PLoS ONE.

[226] Kuvarzin S.R., Sukhanov I., Onokhin K., Zakharov K., Gainetdinov R.R. (2023). Unlocking the Therapeutic Potential of Ulotaront as a Trace Amine-Associated Receptor 1 Agonist for Neuropsychiatric Disorders. Biomedicines.

[227] Alnefeesi Y., Sukhanov I., Gainetdinov R.R. (2024). Ligands of the trace amine-associated receptors (TAARs): A new class of anxiolytics. Pharmacol. Biochem. Behav..

[228] Tseilikman D.M.V., Tseilikman V.E., Shatilov V.A., Obukhova D.A., Zhukov I.S., Yatsyk I.V., Maistrenko V.A., Shipelin V.A., Trusov N.V., Karpenko M.N. (2025). New Insights into Complex PTSD Treatment: Focus on TAAR1 Agonists. Biomedicines.

[229] Alnefeesi Y., Murtazina R.Z., Gainetdinov R.R. (2025). Trace Amine-associated Receptors (TAARs): Candidate Targets in the Treatment of Bipolar Disorders. Actas Esp. Psiquiatr..

[230] Shemiakova T.S., Efimova E.V., Gainetdinov R.R. (2024). TAARs as Novel Therapeutic Targets for the Treatment of Depression: A Narrative Review of the Interconnection with Monoamines and Adult Neurogenesis. Biomedicines.

[231] Sharma O., Grewal A.K., Kumar A., Singh V., Singh T.G., Singh T., Ahmad S.F., Al-Mazroua H.A. (2025). CREB-dependent neurobehavioral alterations via TAAR1 receptor modulation in a chronic unpredictable stress model. Life Sci..

[232] Polini B., Ricardi C., Bertolini A., Carnicelli V., Rutigliano G., Saponaro F., Zucchi R., Chiellini G. (2023). T1AM/TAAR1 System Reduces Inflammatory Response and β-Amyloid Toxicity in Human Microglial HMC3 Cell Line. Int. J. Mol. Sci..

[233] Dalvi S., Bhatt L.K. (2025). Trace amine-associated receptor 1 (TAAR1): An emerging therapeutic target for neurodegenerative, neurodevelopmental, and neurotraumatic disorders. Naunyn. Schmiedebergs. Arch. Pharmacol..

[234] Bryant R.A. (2019). Post-traumatic stress disorder: A state-of-the-art review of evidence and challenges. World Psychiatry.

[235] Schumacher S., Niemeyer H., Engel S., Cwik J.C., Laufer S., Klusmann H., Knaevelsrud C. (2019). HPA axis regulation in posttraumatic stress disorder: A meta-analysis focusing on potential moderators. Neurosci. Biobehav. Rev..

[236] Shi Y., Zhao T., Peng L., Wang P., Huang X., Xu F., Tan Y., Peng S., Xiong T., Bai Y. (2025). Investigating the shared genetic architecture between post-traumatic stress disorder and neurodegenerative diseases: A large-scale genomewide cross-trait analysis. Int. J. Surg..

[237] Trist B.G., Hare D.J., Double K.L. (2019). Oxidative stress in the aging substantia nigra and the etiology of Parkinson’s disease. Aging Cell.

[238] Lee D.H., Lee J.Y., Hong D.Y., Lee E.C., Park S.W., Lee M.R., Oh J.S. (2022). Neuroinflammation in Post-Traumatic Stress Disorder. Biomedicines.

[239] Bonomi R., Hillmer A.T., Woodcock E., Bhatt S., Rusowicz A., Angarita G.A., Carson R.E., Davis M.T., Esterlis I., Nabulsi N. (2024). Microglia-mediated neuroimmune suppression in PTSD is associated with anhedonia. Proc. Natl. Acad. Sci. USA.

[240] Ferreira S.A., Romero-Ramos M. (2018). Microglia response during Parkinson’s disease: Alpha-synuclein intervention. Front. Cell. Neurosci..

[241] Forloni G. (2023). Alpha Synuclein: Neurodegeneration and Inflammation. Int. J. Mol. Sci..

[242] Ressler K.J., Berretta S., Bolshakov V.Y., Rosso I.M., Meloni E.G., Rauch S.L., Carlezon W.A. (2022). Post-traumatic stress disorder: Clinical and translational neuroscience from cells to circuits. Nat. Rev. Neurol..

[243] Smoller J.W. (2016). The Genetics of Stress-Related Disorders: PTSD, Depression, and Anxiety Disorders. Neuropsychopharmacology.

[244] Lee J.C., Wang L.P., Tsien J.Z. (2016). Dopamine Rebound-Excitation Theory: Putting Brakes on PTSD. Front. Psychiatry.

